# An update on global mining land use

**DOI:** 10.1038/s41597-022-01547-4

**Published:** 2022-07-22

**Authors:** Victor Maus, Stefan Giljum, Dieison M. da Silva, Jakob Gutschlhofer, Robson P. da Rosa, Sebastian Luckeneder, Sidnei L. B. Gass, Mirko Lieber, Ian McCallum

**Affiliations:** 1 Vienna University of Economics and Business (WU), Institute for Ecological Economics, Vienna, 1020 Austria; 2 International Institute for Applied Systems Analysis (IIASA), Advancing Systems Analysis Program, Laxenburg, A-2361 Austria; 3 Federal University of Pampa (UNIPAMPA), Itaqui, 97650-000 Brazil; 4 Federal University of Santa Maria (UFSM), Polytechnic College, Santa Maria, 97105-900 Brazil

**Keywords:** Environmental impact, Environmental impact, Sustainability, Environmental economics

## Abstract

The growing demand for minerals has pushed mining activities into new areas increasingly affecting biodiversity-rich natural biomes. Mapping the land use of the global mining sector is, therefore, a prerequisite for quantifying, understanding and mitigating adverse impacts caused by mineral extraction. This paper updates our previous work mapping mining sites worldwide. Using visual interpretation of Sentinel-2 images for 2019, we inspected more than 34,000 mining locations across the globe. The result is a global-scale dataset containing 44,929 polygon features covering 101,583 *km*^2^ of large-scale as well as artisanal and small-scale mining. The increase in coverage is substantial compared to the first version of the dataset, which included 21,060 polygons extending over 57,277 *km*^2^. The polygons cover open cuts, tailings dams, waste rock dumps, water ponds, processing plants, and other ground features related to the mining activities. The dataset is available for download from 10.1594/PANGAEA.942325 and visualisation at www.fineprint.global/viewer.

## Abstract (summary)

**Table:** 

Measurement(s)	terrestrial mining
Technology Type(s)	satellite imaging
Factor Type(s)	area • polygon geometry
Sample Characteristic - Environment	land
Sample Characteristic - Location	Earth (planet)

## Background & Summary

Driven by the growing global demand for raw materials^1^, mineral extraction has expanded particularly into biodiversity-rich ecosystems in the past two decades^2^, and demand trends are projected to further increase^3,4^. Mining can cause a wide range of adverse impacts during mining operation and after closure, e.g. fragmenting the landscape and polluting soils and water with effects on human settlements, agriculture plantations, and natural ecosystems^5^. Mapping the global mining areas is increasingly important for quantifying pressures of mineral extraction on biodiversity^6–9^, land-use modelling^10^, estimating the impacts of global supply chains and sustainable resource use^11–13^, for risk assessments of major environmental disasters on mining areas^14,15^, and planning and reinforcing mine reclamation^16^.

The increasing availability of high-resolution Earth observation data and new machine learning approaches has allowed mapping and monitoring of mining land use and its related environmental impacts on a local or regional scale^17,18^. However, automatically mapping mining areas on a global scale is challenging because they are composed of a set of heterogeneous land cover types^17^. Mining areas are used for various purposes, including the mine itself (e.g. open cuts where the minerals are extracted), waste dumps (e.g. tailings dams, waste rock piles), water ponds, and industrial processing facilities. Additionally, different minerals (e.g. coal, copper, or gold), extraction and processing methods, and landscape characteristics also increase intraclass variability, challenging automated mapping approaches using Earth observation data on a large scale.

Visual interpretation of high-resolution satellite images has been used as an alternative to producing three global mining land use datasets. The first dataset mapped the 295 major mine sites worldwide, adding a total area of 3,633 *km*^2^ ^19^. The second data source mapped a total area of 31,396 *km*^2^ including active and inactive mining sites^20^ and the third dataset, described in our previous article^21^, covered 6,201 active mining sites that add to 57,277 *km*^2^. These three datasets are not comparable because they were derived using different satellite data sources acquired at different times and with distinct spatial resolutions. In addition, each dataset covered a different subset of mining locations, which can lead to underestimating the global mining land use because subnational mining activities are usually underreported compared to national accounts^2,22^.

Here we present a new dataset that improves global mining land use accounting by significantly expanding our previous global-scale dataset of mining sites^21,23^. The data update includes 44,929 polygon features covering 101,583 *km*^2^ of large-scale mining (LSM) as well as artisanal and small-scale mining (ASM). We followed a similar methodology based on visual interpretation to map all 34,820 mining coordinates reported in the SNL Metals & Mining database^24^. Compared to the first version, this is a substantial expansion, which covered only 6,201 coordinates of mines reported as active in the SNL database. As in the previous version, we mapped all land cover types related to mining without distinguishing them within the polygons. Although significantly expanded, our dataset still does not cover all existing mines worldwide, as we only inspected areas within a 10 *km* buffer around the coordinates from SNL^24^. However, to date, our updated dataset provides the most comprehensive information on global mining land use, including openly available georeferenced mining locations.

## Methods

Version 2 of the global-scale mining area dataset builds on the polygons from the first data release^23^ and follows a similar methodology. We updated the areas in the first version using satellite images from 2019 and added new areas not included in the previous version. We inspected all 34,820 coordinates reported in the SNL database, substantially expanding the coverage compared to Version 1, which covered only 6,201 coordinates of mines reported with the status “active” or having any reported production between 2000 and 2017 by SNL^21^. We inspected all SNL coordinates in the second version because several SNL locations with “inactive” status and no reported production have clear ongoing mining activities visible in satellite images. Therefore, inspecting all SNL coordinates independently from their reported status was critical to provide a more comprehensive overview of the global mining land use. This data update also improved the coverage of ASM areas, which were almost absent from the first version because most ASM activities do not report production or activity in the SNL database, although their approximate coordinates are reported.

### Study area

To make the visual interpretation of images viable on a global scale, we limited the area of inspection to a 10 *km* buffer around the coordinates in the SNL database. Based on our previous experience^21^, this buffer size is sufficient to cover large mining sites expanding over several kilometres and also takes into account the imprecision in the SNL coordinates that can be up to 3 *km* distant from the actual mining sites^7,8^. We mapped all mines identified inside or intersecting the buffers’ borders, including areas that start inside the buffer and extend beyond its limits. This protocol was adopted to make sure mines that extend over long distances would be well captured, e.g. ASM mining following deposits on rivers and streams.

### Mining areas

We defined mining areas as all land used by the mining sector at any step in extraction and processing at the mining site. Our mining areas also cover all 111 different commodities reported in the SNL database, including primary and companion commodities (see the complete list of commodities in Table 1). This definition includes different ground features, such as open cuts, tailings dams, waste rock dumps, water ponds, processing plants, and other infrastructure used in LSM and ASM activities. We mapped all underground and above-ground mining infrastructure visible on the satellite images. We did not distinguish between the different infrastructure types, i.e. we aggregated them into a single mining land-use class that includes all the above-mentioned ground features. Following this approach, we produced a global dataset with the georeferenced extent of mining land use that can be used as a starting point to distinguish LSM and ASM and their different infrastructure types in future work.

**Table 1:** List of all commodities reported in the SNL database.

Commodity name (Number of mines reporting the commodity)		
Gold (17526)	Yttrium (69)	Lutetium (9)
Copper (8699)	Potassium Oxide (68)	Thulium (9)
Silver (7215)	Barite (53)	Borates (8)
Coal (5164)	Rhenium (47)	Erbium (8)
Zinc (4168)	Scandium (47)	Holmium (8)
Lead (3337)	Magnesium (44)	Limestone (7)
Iron Ore (2280)	Iridium (39)	Osmium (7)
U3O8 (2013)	Leucoxene (39)	Selenium (7)
Nickel (1951)	Thorium (38)	Alumina (6)
Diamonds (1515)	Cadmium (36)	Hafnium (6)
Molybdenum (1461)	Ruthenium (36)	Beryl (5)
Cobalt (1079)	Caesium (35)	Ferrochrome (5)
Platinum (1024)	Indium (33)	Ferrovanadium (5)
Palladium (972)	Tellurium (31)	Gypsum (5)
Rhodium (578)	Beryllium (29)	Aggregates (4)
Lanthanides (533)	Spodumene (29)	Aluminum (4)
Lithium (490)	Chromium (26)	Sapphire (4)
Tungsten (424)	Cerium (25)	Strontium (4)
Tin (419)	Neodymium (25)	Emerald (3)
Manganese (353)	Iron Sand (24)	Ferrotungsten (3)
Graphite (333)	Rare Earth Elements (24)	Kaolin (3)
Phosphate (325)	Rubidium (23)	Calcium Carbonate (2)
Magnetite (295)	Mercury (22)	Hematite (2)
Vanadium (290)	Gallium (20)	Jade (2)
Potash (262)	Lanthanum (20)	Platinum Group Metals (2)
Tantalum (242)	Praseodymium (20)	Potassium Sulfate (2)
Bauxite (241)	Dysprosium (17)	Topaz (2)
Chromite (217)	Germanium (14)	Vermiculite (2)
Titanium (191)	Silica (14)	Asbestos (1)
Antimony (190)	Terbium (14)	Boron (1)
Ilmenite (181)	Europium (13)	Ferromanganese (1)
Niobium (174)	Samarium (13)	Frac Sand (1)
Zircon (171)	Garnet (12)	Heavy Rare Earths and Yttrium (1)
Rutile (152)	Potassium Chloride (11)	Marble (1)
Heavy Mineral Sands (150)	Gadolinium (10)	Promethium (1)
Bismuth (99)	Ytterbium (10)	Ruby (1)
Arsenic (70)	Ferronickel (9)	Sodium Bicarbonate (1)

### Delineation of mining areas

The new version of the data set significantly improved temporal consistency. In the previous version, we used images from Google Earth imagery, Microsoft Bing Imagery and Sentinel-2 cloudless^25^. However, Google Satellite and Microsoft Bing Imagery provide heterogeneous spatial resolution across the globe, and in many areas, their images are outdated by several years^26^. For the update, we delineated the areas always using the 2019 Sentinel-2 cloudless mosaic, which provides homogeneous 10 m spatial resolution and a well-defined time frame for the entire globe^25^. We only consulted Google Earth and Microsoft Bing for additional information in case of doubt about a ground feature but did not use these images to delineate the mines.

All three satellite data sources were visually inspected using our open-source web application^27^ developed for this specific purpose. The web interface systematically displays buffers and markers with information about the mines, which were used to limit the study area and to provide additional information about mining types and commodities. After visually inspecting all satellite data sources, the interpreter delineated the mining areas using Sentinel-2 cloudless^25^ as the background layer. Note that we did not map mining features in regions where the quality of the images did not allow proper interpretation. However, only a few of the inspected locations were unclear because the Sentinel-2 cloudless layer by EOX mosaics all acquisitions from one year to produce yearly composites with significantly reduced cloud cover and atmospheric interference^25^.

The mining polygons can also contain isolated patches with forest or other land covers, not necessarily representing any land cover related to mining activity. We included these isolated patches on the mining polygons because they usually do not have other use and have a reduced ecological function as landscape fragmentation reduces the ability of the ecosystem to provide ecosystem services^28^.

It is important to note that we could not keep the relation between the SNL coordinates and the delineated polygons. In most cases, SNL provides several coordinates clustered around a number of mining ground features identified in the satellite images. However, the information from satellite images is not sufficient to link these features with the SNL coordinates without additional fieldwork. Besides that, some mines displace waste dumps and other infrastructure several kilometres from the main mining site, making it difficult to confidently link them to the coordinates using only information from satellite images. Therefore, our methodology uses the SNL coordinates only to gather information on the locations where mining might occur, but our final data product does not include information or links to the SNL database such as coordinates, commodities or production volumes.

### Geoprocessing of data records

The delineated mining areas produced a raw data collection of polygons, which were checked and corrected by geoprocessing operations in R using the packages sf^29^ and s2^30^. We removed the double-counting of mining areas by uniting overlapping polygons and corrected all invalid geometries, for example, due to crossing edges accidentally created during the digitalisation of the polygons. After that, we removed *sliver polygons* (unwanted small polygons) and polygons with persistent invalid geometries, finally producing a consistent set of polygons simple features^29^.

We then calculated the area of each feature and added information on the country in which each polygon is located. We calculated the area in square kilometres using spherical geometry^30^. After that, a spatial join query acquired country names and ISO 3166-1 alpha-3 codes from the country’s administrative units geometries available from EUROSTAT^31^. The final set of polygons thus includes the geometries (polygons) covering the mining areas, their respective areas in square kilometres, country name, and ISO 3166-1 alpha-3 code of the corresponding country.

Similarly to Version 1, we also derived global grid datasets with the mining area at 30 *arcsecond*, 5 *arcminute* and 30 *arcminute* spatial resolution (approximately 1 × 1 *km*, 10 × 10 *km* and 50 × 50 *km* at the equator). This is useful as many modelling applications require regular grid data^32^. The 30 *arcsecond* grid was derived from the percentage of the polygons’ area intersecting each cell. The percentages were rounded to zero decimal digits to reduce the size of the dataset. Therefore, the percentage of mining area covering a cell should be greater than 0.5% to be considered, i.e., approximately 0.5 *ha* at the equator. To obtain the gridded mining area, we estimated the area of each cell in square kilometres and multiplied it with the percentage of mining cover per cell, resulting in a 30 *arcsecond* global grid indicating the mining area within each cell. The other two grid levels, 5 *arcminute* and 30 *arcminute*, were resampled from the 30 *arcsecond* grid. The scripts used in the geoprocessing of data records are available with our open-source web application tool^27^.

## Data Records

The new dataset consists of 44,929 polygon features covering 101,583 *km*^2^ of mining areas worldwide^33^. It more than doubles the number of polygons compared to Version 1 (21,060 polygons) and nearly doubles the mapped area, previously 57,277 *km*^2^ ^21^. The number of countries covered also increased from 121 to 145. Besides the polygons, grid data provides a ready-to-use dataset for modelling with the mining area in square kilometres per grid cell provided at 30 *arcsecond*, 5 *arcminute*, and 30 *arcminute* spatial resolution. All data records were deposited to PANGAEA (Data Publisher for Earth & Environmental Science) and are available from 10.1594/PANGAEA.942325. The data is also available for visualisation from our platform www.fineprint.global/viewer. In what follows, we present a few examples to illustrate the data and provide an overview of the global mining land use compared to the first version of the data.

### Examples of mapped areas

**Fig. 1:**
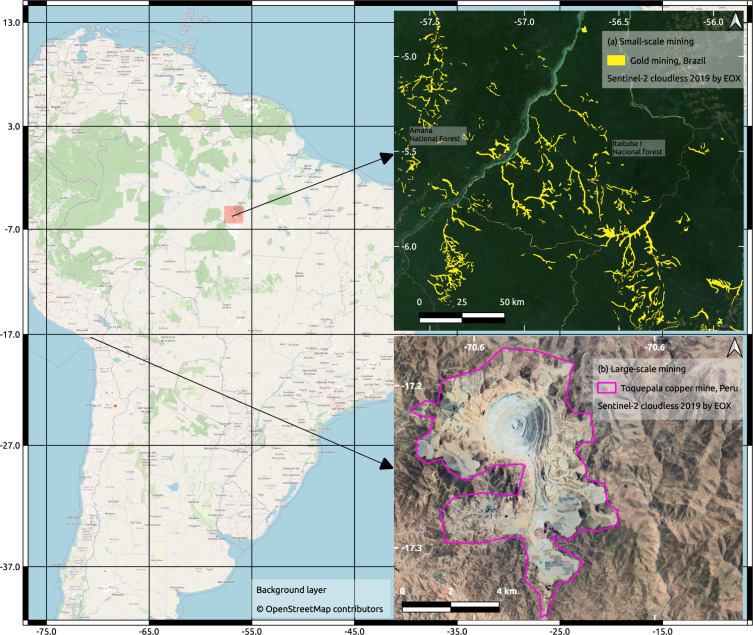
Mapped small- and large-scale mining in South America. (**a**) Small-scale gold mining in the Brazilian Amazon on both sides of the Tapajós River in the Brazilian state of Pará. (**b**) Toquepala copper mine in Tacna Province, Peru.

The maps in Fig. 1 show examples of LSM and ASM. The map in the top right of Fig. 1 illustrates the spatial pattern of ASM gold mining in the Brazilian Amazon. In this region, mining activities can spread over hundreds of kilometres, usually following water streams^34^. The same spatial pattern can be found in other areas worldwide, such as in Ghana^35^. In the bottom right of Fig. 1 we illustrate LSM areas with an example of the Toquepala copper mine in Peru. We invite the reader to explore other regions in our web platform at www.fineprint.global/viewer.

### Global mining land use

**Fig. 2:**
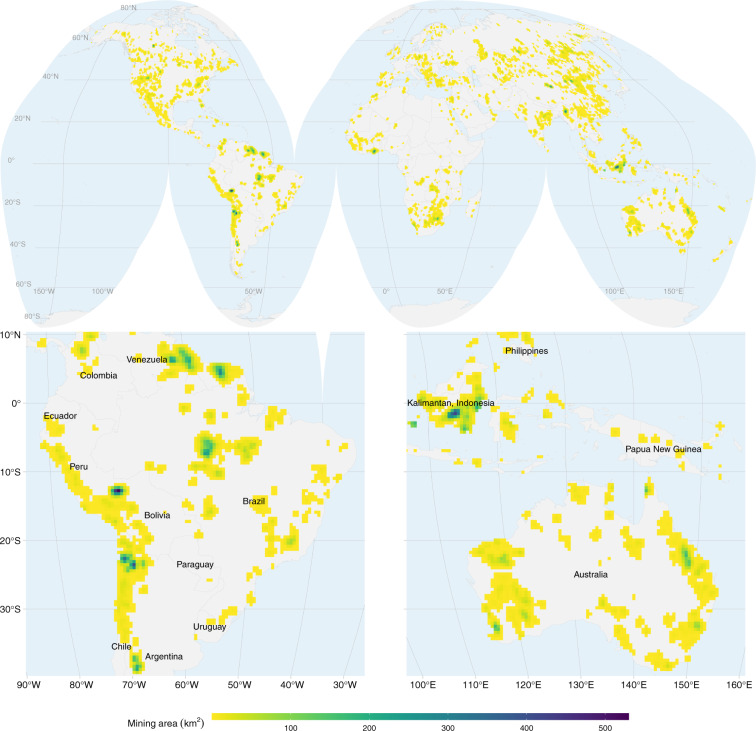
Global overview of mining areas mapped in Version 2 aggregated to 5050 *km* grid cells and projected to Interrupted Goode Homolosine. The maps at the bottom are zoomed to South America (left), and Australia and parts of South-East Asia (right).

Figure 2 shows the geographical distribution of the mining area across the globe. The map in the figure is projected to equal area Interrupted Goode Homolosine and the mining areas resampled to a 50 × 50 *km* grid to facilitate visualisation. Except for Antarctica, mining spreads across all continents with some hot-spot regions, for example, in northern Chile mainly due to copper extraction, northeastern Australia and East Kalimantan in Indonesia because of coal mining, and in the Amazon rain forest primarily due to small-scale gold mining.

**Fig. 3:**
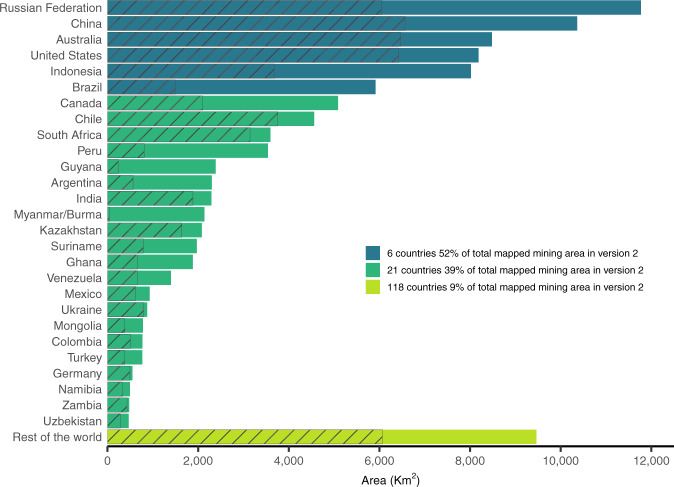
Mining land use per country in square kilometres. The dashed bars indicate the areas mapped in Version 1 of the dataset.

A summary of our data aggregated by country shows that 52% of the mapped mining area is concentrated in only six countries: Russia, China, Australia, the United States, Indonesia, and Brazil. Another 21 countries account for 39%, and the remaining 118 countries add up to only 9% of the total mapped mining area (see Fig. 3). These results show that mining areas are highly concentrated in only a few countries.

Compared to the area mapped in Version 1 of the dataset^23^ (dashed bars in Fig. 3), we see that the ranking of countries has changed. Russia, for instance, held the fourth position in the first version, but is the country with the largest mining land use in Version 2. The large difference is due to the substantial increase in the number of regions visually inspected, including the buffer around all coordinates reported in the SNL database independently from their activity status or reported production. This allowed us to identify ongoing mining activities from the satellite images in many regions with no reported production and to significantly improve the coverage of global mining land use. The substantially larger area mapped in Version 2 (nearly double the area mapped in Version 1), also indicates that mineral extraction amounts are underreported in the SNL database. This can have implications for studies that rely on SNL’s production data and urges for more transparency on the quantities of material extracted in mines worldwide.

**Fig. 4:**
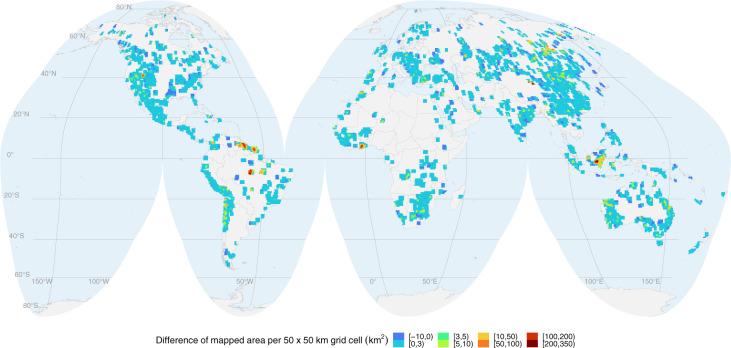
Global overview of additional mining area mapped in Version 2 compared to Version 1, aggregated to 5050 *km* grid cells and projected to Interrupted Goode Homolosine.

Figure 4 highlights the spatial distribution of the difference in the area mapped in Version 2 compared to Version 1 within a 50 × 50 km grid. Most grid cells increased their mapped area between three and five square kilometres. Some regions also reduced the mining area from Version 1 to Version 2. However, this decrease was not caused by abandoned mine sites nor rehabilitation, but it is an artefact of the more accurate delineation of the borders of the polygons in Version 2. In the map, we can also note a few hotspots with a substantial increase in the mining area, e.g. Brazil, Guyana, Suriname, Ghana, and Indonesia, mostly due to the better coverage of ASM on river and water streams in Version 2.

Table 2 presents a summary of the area and number of polygons per country, illustrating different profiles of countries regarding the spatial distribution of the mines. For example, Russia and China have comparable figures regarding total mapped mining areas, 11,770.93 *km*^2^, and 10,364.57 *km*^2^. However, the number of identified polygons in China was significantly higher than in Russia, 8,795 against 2,825. This indicates structural differences in the mining sectors, i.e. a larger number of mining areas of smaller size in China compared to Russia, highlighting the known presence of a small-scale mining industry in China^36,37^.

**Table 2:** Mining area in *km*^2^ and the number of polygons (n) mapped per country. The countries are indicated by their respective ISO 3166-1 alpha-3 code.

Country	*km*^2^	n	Country	*km*^2^	n	Country	*km*^2^	n
RUS	11,770.93	2,825	CZE	165.88	73	ETH	16.30	33
CHN	10,364.57	8,795	SWE	159.29	219	CYP	16.27	32
AUS	8,482.63	3,416	SRB	149.67	192	PAN	15.57	21
USA	8,188.54	3,899	TZA	146.60	225	LBR	15.13	51
IDN	8,020.15	1,448	FIN	143.90	288	AUT	14.94	48
BRA	5,915.79	2,427	NER	127.52	35	GEO	14.60	9
CAN	5,087.56	2,828	KGZ	126.61	105	URY	13.45	28
CHL	4,562.65	697	MOZ	123.42	72	JPN	13.22	47
ZAF	3,594.62	1,526	CUB	118.32	65	TKM	13.07	3
PER	3,539.54	852	NZL	118.08	181	LSO	12.20	9
GUY	2,388.75	456	MRT	114.89	43	MNE	10.87	13
ARG	2,301.00	334	BFA	112.51	89	ERI	10.63	6
IND	2,293.41	1,204	MYS	112.12	118	GTM	10.60	20
MMR	2,140.09	170	SAU	111.40	74	COG	10.37	26
KAZ	2,082.59	656	CIV	107.30	44	LKA	10.12	45
SUR	1,972.02	306	SLE	88.51	104	HND	9.66	16
GHA	1,882.81	577	PNG	77.30	38	IRQ	8.85	4
VEN	1,401.40	105	TUN	75.48	25	AFG	6.79	10
MEX	932.22	1,583	EGY	72.75	29	SLB	5.93	3
UKR	877.10	707	HUN	71.39	110	TGO	5.43	3
MNG	782.92	429	ECU	71.02	97	MWI	5.00	14
COL	772.43	219	ESH	62.91	3	KHM	4.60	16
TUR	769.49	911	SEN	58.96	27	CMR	4.41	5
DEU	550.87	146	LAO	56.49	56	ARE	4.21	6
NAM	494.68	262	ISR	55.26	10	FJI	3.53	18
ZMB	480.05	149	MKD	53.57	34	PRY	3.52	29
UZB	468.39	73	GAB	51.84	13	HTI	3.36	10
COD	426.22	223	SDN	50.95	33	UGA	3.14	17
MAR	369.73	96	MDG	44.47	63	BEL	1.91	3
IRN	363.08	167	ARM	43.40	71	SVN	1.75	4
POL	331.66	218	DZA	42.77	97	RWA	1.64	16
BWA	315.20	171	PRT	39.94	162	LUX	1.42	5
PHL	302.68	350	KOR	39.11	125	NLD	1.42	8
AGO	294.51	218	NOR	35.88	109	CRI	1.36	4
ESP	292.44	256	TJK	34.23	67	BGD	1.27	2
BOL	286.77	138	DOM	31.18	29	SJM	1.03	3
JOR	263.89	46	BIH	29.06	14	SOM	0.70	3
VNM	263.31	146	IRL	25.81	97	SLV	0.59	5
NCL	251.66	158	JAM	25.62	56	CHE	0.55	10
ZWE	242.48	320	BLR	24.69	6	GRL	0.36	2
GIN	231.34	128	NGA	24.65	70	ABW	0.36	2
BGR	226.15	109	AZE	24.59	25	SWZ	0.33	2
GRC	216.26	73	PRK	24.52	30	TCD	0.20	2
OMN	201.53	110	KEN	23.52	29	BEN	0.11	2
FRA	199.46	158	ITA	23.30	76	BDI	0.08	1
MLI	194.94	78	PAK	22.80	28	GNB	0.06	2
ROU	176.98	94	SVK	19.43	94	ISL	0.05	1
THA	168.98	81	ALB	17.20	91			
GBR	168.91	203	NIC	16.98	28			
**Total Area: 101,583.4 *km***^**2**^**; Polygons: 44,929; Countries: 145**								

## Technical Validation

The mapping work was performed by trained interpreters exclusively using satellite images. Most mining areas are identifiable in the satellite images for the human eye. However, some areas can be challenging to interpret, creating a source of commission (no-mine areas mapped as mines) and omission errors (mine areas not mapped as mines). Besides that, the borders of the mines are not always evident in the images, creating another source of uncertainty.

We performed an independent classification of random points to assess these mapping errors. We followed the best practices on map accuracy assessment and sample design for overall accuracy, user’s accuracy (or commission error), and producer’s accuracy (or omission error)^38^. We drew a set of 1,220 random points stratified between the area mapped as mine and those not mapped as mine (no-mine) within the region of interest (10 *km* buffer from the geographical coordinates). These validation points were inspected independently by experts that did not participate in the delineation of the mines. They classified these validation points as mine or no-mine based on the satellite data without information on whether the points were mapped as part of a mining area. The validation points are also available from the data record^33^.

Based on these control points, we provide a range of assessment metrics. The overall accuracy shows that 88.3% of the control points were correctly classified, and the high F1 score of 0.87 indicates a low penalisation for false negatives^39^. The Kappa index was 0.77 and Matthews correlation coefficient (MCC) 0.78 (Kappa and MCC range from −1 to 1^40^). Negative values imply that the agreement is worse than random; 1 presents a complete agreement, while 0 is the expected value for a random classification). Our dataset also had an 89.7% probability of correctly distinguishing mining from non-mining areas according to the area under the curve (AUC) of the Receiver Operating Characteristic (ROC) curve^41^. We also derived the user’s and producer’s accuracy along with the error matrix (see Table 3) as recommended in map accuracy assessment^38,42^. The user’s accuracy tells how well the classes in the map represent the reality on the ground, while the producer’s accuracy points to how well a class has been mapped^38^. Our map reached a 78.9% producer’s accuracy, indicating that we missed some mining areas (the omission of mines was around 21.2% in our validation samples). However, the mapped mining areas had 97.2% user’s accuracy, i.e. the mapped mining areas have a high probability of being correctly mapped as mining (less than 3% incorrectly mapped as mining).

**Table 3:** Error matrix and accuracy statistics derived from 1,220 random points equally allocated between the mapped classes Mine and No-mine.

Mapped	Reference			User’s acc. (%)
	Mine	No-mine	Total	
Mine	481	129	610	97.2
No-mine	14	596	610	82.2
Total	495	725	1220	
Producer’s acc. (%)	78.9	97.7		
Overall acc.: 88.3%; Kappa: 0.77; F1 Score: 0.87; MCC: 0.78				

We also investigated whether the proximity to the borders of the mines has affected the accuracy. We found that 54.5% of the control points with disagreement are located less than 50 *m* from the borders of the delineated polygons. On the other hand, only 16% of points with an agreement are located closer than 50 *m* to the polygons’ borders. These results indicate that higher uncertainty lies closer to the borders of the mapped areas. Additionally, it indicates high confidence in the existence of mines within the mapped polygons.

## Usage Notes

The global mining dataset described here is available from 10.1594/PANGAEA.942325 under the Creative Commons Attribution-ShareAlike 4.0 International (CC-BY-SA) license. The data records include the same resources as the previous data release^23^ – the mining polygons, validation points, mining area grid, and a summary per country’s mining area.

1. The mining polygons and validation points are encoded in *GeoPackage* geographic data structures^43^, such that:
  - the *mining_polygons* layer has five attributes:
    ISO3_CODE: A string with the country’s ISO 3166-1 alpha-3 code
    COUNTRY_NAME: A string with the country name in English
    AREA: A number with the area of the feature in square kilometres
    geom: A polygon geometry in geographical coordinates WGS84
    fid: An integer with feature ID
  - the *validation_points* layer has four attributes:
    MAPPED: A string with the class derived from the mining polygons (“mine” or “no-mine”)
    REFERENCE: A string with the validation class (“mine” or “no-mine”)
    geom: A point geometry in geographical coordinates WGS84
    fid: An integer with feature ID
2. The mining grids include a single layer each (one band raster) encoded in Geographic Tagged Image File Format (GeoTIFF)^44^. Each grid cell over land has a float number (data type *Float32*) greater than or equal to zero representing the mining area in square kilometres; grid cells over water have no-data values. The grid is available in three spatial resolutions, 30 *arcsecond*, 5 *arcminute*, and 30 *arcminute*, extending from the longitude −180 to 180 degrees and from the latitude −90 to 90 degrees in the geographical reference system WGS84.
3. The summary of the mapped mining area per country derived from the mining polygons is available in Comma-separated values (CSV)^45^ format, including four attributes:
  COUNTRY_NAME: A string with the country name in English
  ISO3_CODE: A string with the country ISO3 code
  AREA: A number with the area of the feature in square kilometres
  N_FEATURES: An integer with the number of features per country

The datasets can easily be overlaid with other geospatial variables for further spatial analysis using software with support Geographic Information System (GIS) (e.g. including QGIS^46^, R^47^, and Python^48^). Besides, we also provide a tool for visual analysis of the geographical data records at www.fineprint.global/viewer and a Web Map Service (WMS)^49^ accessible from www.fineprint.global/geoserver/wms.

## Data Availability

All the code and geoprocessing scripts used to produce the results of this paper are distributed under the GNU General Public License v3.0 (GPL-v3)^50^ from the repository www.github.com/fineprint-global/app-mining-area-polygonization^27^. The processing scripts were written in R^47^, Python^48^, and GDAL (Geospatial Data Abstraction Library^51^). The web application to delineate the polygons was written in R Shiny^52^ using a PostgreSQL^53^ database with PostGIS^54^ extension for storage. The full app setup uses Docker^54^ containers to facilitate management, portability, and reproducibility.
